# Proteome data of serum samples from patients with schizophrenia

**DOI:** 10.1016/j.dib.2020.105338

**Published:** 2020-02-25

**Authors:** T.V. Butkova, A.T. Kopylov, A.A. Stepanov, K.A. Malsagova, G.P. Kostyuk, N.V. Zakharova, L.V. Bravve, A.A. Sinicyna, A.L. Kaysheva

**Affiliations:** a V. N. Orekhovich Research Institute of Biomedical Chemistry, Moscow, Russia; b State Healthcare Institution «Psychiatric Clinical Hospital 1 n. a. N.A. Alekseev of Healthcare Department of Moscow», Russia

**Keywords:** Serum, Schizophrenia, Proteomics, Tandem mass spectrometry

## Abstract

Schizophrenia is a complex chronic disease. The molecular determinants and neuropathology of schizophrenia are multifaceted; an important role in the pathogenesis is played by the dysregulation of molecular and epigenetic mechanisms. However, the molecular mechanisms of the development of the disease have not yet been studied.

An important task is the accumulation and systematization of “OMICS”-knowledge of the molecular profiles (transcriptome, proteome, metabolome) of blood specific to pathology. Thereby the development and improvement of mass spectrometric methods for the detection of biological molecules has become increasingly important in biomedical research. In the field of applied problems in biomedical research, the most prevalent issue involves the identification of serological protein markers associated with the development of schizophrenia, which account for the diseases that cause the a life-shortening illness, disability, decreased of functioning and quality of life and wellbeing or health status.

OMICS approaches are designed to detect genes (genomics), mRNA (transcriptomics), proteins (proteomics) and metabolites (metabolomics) in a specific biological sample.

We report the proteomic datasets on the serum samples from patients with schizophrenia (series “SCZ”) and healthy volunteers (series “CNT”). Data were acquired using shotgun ultra-high resolution mass spectrometry.

**Table:** Specifications Table

Subject	Biology
Specific subject area	Biochemistry, omics analysis, protein detection
Type of data	Table, Text file
How data were acquired	Liquid chromatography-tandem mass spectrometric analysis was carried out using Q Exactive high-resolution mass spectrometer (Thermo Fisher Scientific, USA) coupled with an Ultimate 3000 Nano-flow HPLC system (Thermo Fisher Scientific, USA)
Data format	Raw, filtered
Parameters for data collection	50 control samples blood serum from healthy volunteers and 49 samples from blood serum from patients with schizophrenia
Description of data collection	- Digestion of proteins. - LC-MS/MS analysis. - Data processing.
Data source location	Moscow, Russia
Data accessibility	Proteomic data have been deposited to the ProteomeXchange Consortium via the PRIDE partner repository with the dataset identifier PXD016297. https://www.ebi.ac.uk/pride/archive/projects/PXD016297

**Table:** 

**Value of the Data** • Dataset represents proteomes of serum samples from patients with schizophrenia as well as from control healthy volunteers, which can be compared to reveal molecular pathways of pathology. • Blood plasma serves as an attractive source of candidate protein markers and specific pathologies for molecular profiling, as it contains molecular components secreted by cells in diseased tissues, as well as factors involved in the development of pathophysiological processes. • Protein profiling are perspective to reveal for clinical monitoring of drug therapy, for identification of affected signalling pathways may indicate the direction of research for the development of a systematic approach to the diagnosis and classification of schizophrenia disease. • Protein profiles are available in the form of “∗.raw” and “∗.mgf” data that can be further processed by researchers using their own bioinformatics algorithms and analysed together with their own data.

## 1 Data description

The dataset contains “∗.raw ” and “∗.mgf ” data obtained through the shotgun HPLC-MS/MS analysis of serum samples from 49 patients with schizophrenia and 50 healthy volunteers. Data are available via ProteomeXchange with identifier PXD016297 [1]. Information about blood samples collected from patients with schizophrenia and control samples from healthy donors is presented in Table 1. Dataset covers 99 biological samples (see Table 2).

**Table 1:** Data of schizophrenia patients.

Parameter	Patients, n = 49	Healthy volunteers, n = 50
Male	26	19
Female	23	31
*Family status*		
Married	6	15
Divorced	3	3
Never married	40	32
*Education*^a^		
Incomplete secondary education	2	1
Secondary education	8	15
Secondary special education	13	1
Incomplete higher education	7	4
Higher education	19	29
*Worker status*		
Student	6	37
Working	8	13
Unemployment	10	0
Disabled	0	0
*Average age, years*		
at the time of blood sampling	27,0 ± 5,1	25,9 ± 5,8
first appeal	22,2	–
*Diagnostic tools to confirm schizophrenia* (Mann-Whitney *U* Test < 0.05)		
Positive and Negative Syndrome Scale (PANSS)	112,7	33,1
The Bush-Francis Catatonia Rating Scale (BFCRS)	8,0	0
The 4-Item Negative Symptom Assessment (NSA-4)	21,0	0

a Russia's Educational System.

**Table 2:** Sample description.

Sample ID	Files “∗.mgf ”, “∗.raw ”	Size of “∗.mgf ”, MB	Size of ”∗.raw ”, MB	Type of set
YGW735	CNT_20190613_01_YGW735	36,496	299,933	control
KOHYW0	CNT_20190613_02_KOHYW0	32,508	310,499	control
F0TP43	CNT_20190613_03_F0TP43	12,141	354,819	control
SOV2XB	CNT_20190613_04_SOV2XB	55,592	374,428	control
UNRJB0	CNT_20190613_05_UNRJB0	27,143	309,493	control
D06354	CNT_20190613_06_D06354	66,683	375,034	control
XTJKUA	CNT_20190613_07_XTJKUA	20,075	228,465	control
BEZ9HT	CNT_20190613_08_BEZ9HT	62,221	380,846	control
JXR1JH	CNT_20190613_09_JXR1JH	71,333	384,764	control
V1KI56	CNT_20190613_10_V1KI56	65,864	377,322	control
8EUMZS	CNT_20190613_11_8EUMZS	58,062	369,257	control
ODFBCY	CNT_20190613_12_ODFBCY	55,578	376,181	control
CMIJXTK	CNT_20190613_13_CMIJXTK	61,93	371,116	control
50DQE5	CNT_20190613_14_50DQE5	71,957	384,9	control
A6KS1M	CNT_20190613_15_A6KS1M	30,391	310,726	control
ADAAWO	CNT_20190613_16_ADAAWO	17,498	183,466	control
0O78QX	CNT_20190613_17_0O78QX	59,479	370,889	control
4GO4TM	CNT_20190613_18_4GO4TM	74,33	386,428	control
C5WJWQ	CNT_20190613_19_C5WJWQ	31,879	307,852	control
FAGIC5	CNT_20190613_20_FAGIC5	28,488	275,702	control
WML65Y	CNT_20190613_21_WML65Y	84,281	386,867	control
9L82IT	CNT_20190613_22_9L82IT	56,14	369,903	control
6H1B6S	CNT_20190613_23_6H1B6S	20,307	259,611	control
HIH318	CNT_20190613_24_HIH318	27,607	289,802	control
0SFACQ	CNT_20190613_25_0SFACQ	20,354	264,346	control
9U3UGD	CNT_20190613_26_9U3UGD	73,548	376,671	control
PZRMAK	CNT_20190613_27_PZRMAK	26,31	310,059	control
VLXKLO	CNT_20190613_28_VLXKLO	27,886	309,922	control
TANU6P	CNT_20190613_29_TANU6P	17,669	244,21	control
MUM0IX	CNT_20190613_30_MUM0IX	25,266	328,986	control
R6P2S2	CNT_20190613_31_R6P2S2	77,756	379,815	control
KLSD4V	CNT_20190613_32_KLSD4V	61,32	378,474	control
705M82	CNT_20190613_33_705M82	28,658	312,383	control
CLGU1Q	CNT_20190613_34_CLGU1Q	61,026	375,212	control
ZQRSP6	CNT_20190613_35_ZQRSP6	27,541	296,7	control
454ZZV	CNT_20190613_36_454ZZV	3,181	242,924	control
F40I59	CNT_20190613_37_F40I59	28,703	292,511	control
CAQSMO	CNT_20190613_38_CAQSMO	28,1	287,196	control
ZM532 N	CNT_20190613_39_ZM532 N	51,479	371,66	control
50O6VV	CNT_20190613_40_50O6VV	31,519	311,755	control
U9YFIR	CNT_20190613_41_U9YFIR	80,734	371,034	control
C47UZM	CNT_20190613_42_C47UZM	32,161	316,571	control
XJ6412	CNT_20190613_43_XJ6412	34,698	317,005	control
6ZKO63	CNT_20190613_44_6ZKO63	16,174	190,94	control
VMIHKL	CNT_20190613_45_VMIHKL	28,302	285,948	control
OQLRX4	CNT_20190613_46_OQLRX4	31,157	291,616	control
NFP2WG	CNT_20190613_47_NFP2WG	85,756	379,078	control
7CX0U3	CNT_20190613_48_7CX0U3	65,229	372,454	control
HARVQQ	CNT_20190613_49_HARVQQ	52,139	373,033	control
EX6CFH	CNT_20190613_50_EX6CFH	77,873	370,668	control

YC1350	SCH_20190610_01_YC1350	68,356	344,859	schizophrenia
CXB45F	SCH_20190610_02_CXB45F	30,051	256,296	schizophrenia
Z354Y7	SCH_20190610_03_Z354Y7	25,949	272,672	schizophrenia
EFHMWB	SCH_20190610_04_EFHMWB	21,491	272,698	schizophrenia
ZW4912	SCH_20190610_05_ZW4912	47,083	372,386	schizophrenia
YM6648	SCH_20190610_06_YM6648	36,862	349,4	schizophrenia
XO4528	SCH_20190610_07_XO4528	28,831	309,772	schizophrenia
214009	SCH_20190610_08_214009	32,955	329,272	schizophrenia
XT0224	SCH_20190610_09_XT0224	26,24	324,236	schizophrenia
XP2950	SCH_20190610_10_XP2950	30,717	322,526	schizophrenia
XZ8204	SCH_20190610_11_XZ8204	28,852	313,83	schizophrenia
ZL4104	SCH_20190610_12_ZL4104	30,721	300,117	schizophrenia
QL4MJF	SCH_20190610_13_QL4MJF	9,633	419,259	schizophrenia
TW2654	SCH_20190610_14_TW2654	29,695	319,599	schizophrenia
ZM9268	SCH_20190610_15_ZM9268	36,432	331,841	schizophrenia
7T9E6N	SCH_20190610_16_7T9E6N	28,278	306,671	schizophrenia
6BZGDC	SCH_20190610_17_6BZGDC	24,741	294,882	schizophrenia
YN2166	SCH_20190610_18_YN2166	39,257	311,476	schizophrenia
WU2600	SCH_20190610_19_WU2600	23,116	280,623	schizophrenia
ZE3674	SCH_20190610_20_ZE3674	25,114	361,736	schizophrenia
ZT4320	SCH_20190610_21_ZT4320	29,695	297,123	schizophrenia
WZ0609	SCH_20190610_22_WZ0609	26,424	243,559	schizophrenia
XW7605r	SCH_20190610_23_XW7605r	27,021	301,198	schizophrenia
TP9276	SCH_20190610_24_TP9276	32,121	282,769	schizophrenia
XS1165	SCH_20190610_25_XS1165	26,678	311,717	schizophrenia
7DLGDR	SCH_20190610_26_7DLGDR	40,358	351,499	schizophrenia
Z3I4Y7	SCH_20190610_27_Z3I4Y7	26,408	288,453	schizophrenia
XP1143	SCH_20190610_28_XP1143	27,226	333,953	schizophrenia
XR1760	SCH_20190610_29_XR1760	28,219	306,195	schizophrenia
XW9999	SCH_20190610_30_XW9999	30,625	312,772	schizophrenia
YN7802	SCH_20190610_31_YN7802	35,292	300,74	schizophrenia
YM9980	SCH_20190610_32_YM9980	33,33	319,595	schizophrenia
XY2612	SCH_20190610_33_XY2612	35,366	332,174	schizophrenia
1B2AF3	SCH_20190610_34_1B2AF3	31,84	303,574	schizophrenia
KI2824	SCH_20190610_35_KI2824	34,055	308,374	schizophrenia
Z57012	SCH_20190610_36_Z57012	29,843	353,113	schizophrenia
Z00291	SCH_20190610_37_Z00291	32,211	342,074	schizophrenia
YX0920	SCH_20190610_38_YX0920	32,216	351,013	schizophrenia
2B2612	SCH_20190610_39_2B2612	26,518	273,173	schizophrenia
YN3925	SCH_20190610_40_YN3925	25,788	297,709	schizophrenia
GS4953	SCH_20190610_41_GS4953	28,146	314,648	schizophrenia
YT7896	SCH_20190610_42_YT7896	27,905	288,273	schizophrenia
ZB6894	SCH_20190610_43_ZB6894	32,331	300,684	schizophrenia
XW6458	SCH_20190610_44_XW6458	25,98	312,08	schizophrenia
YS2187	SCH_20190610_45_YS2187	37,132	327,972	schizophrenia
ZD0291	SCH_20190610_46_ZD0291	43,1	355,323	schizophrenia
XO6440	SCH_20190610_47_XO6440	28,316	292,074	schizophrenia
XX2845r	SCH_20190610_48_XX2845r	34,265	350,4	schizophrenia
5O9UN9	SCH_20190610_49_5O9UN9	27,288	300,144	schizophrenia

## 2 Experimental design, materials, and methods

### 2.1 Reagents

Acetonitrile and TCA were from Merck (Germany). Formic acid was from ACROS Organics (USA). Ethylenediaminetetraacetic acid (EDTA) was from Sigma-Aldrich (USA). Modified trypsin was from Promega (USA). MOPS (4-morphline-propane-sulphonic acid sodium salt), BUN, deoxycholic acid sodium salt, hydrogen carbonate of ammonium triethyl, ТСЕР (tris-(2-carboxyethil)-phosphine), 4-vinyl pyridine, propane-2 olefins, formic acid (Merck, Germany), deoxycholic acid, methanol, trifluoroacetic acid (Fluka, Germany).

### 2.2 BioSamples

These data include patients with a diagnosis of schizophrenia and healthy controls, all participants signed the informed consent form.

The group of patients: 49 patients (26 men, 23 women, average age 26.9 ± 5.2 years) who were hospitalized in State Healthcare Institution «Psychiatric Clinical Hospital 1 n. a. N.A. Alekseev of Healthcare Department of Moscow» from February to April 2019 with a diagnosis of schizophrenia.

The control group consisted of 50 volunteers from among employees, students and residents who have never sought psychiatric help and who are not related to patients.

### 2.3 Inclusion criteria

1. Age 18 or older
2. Male or female
3. Diagnosis of schizophrenia

The diagnosis of schizophrenia was established on the basis of the criteria of the International Classification of Diseases of the 10th revision (ICD-10) (see Table 3).

**Table 3:** Diagnosis data.

The diagnosis of ICD-10	F20.0	F20.2	F20.8	F21.8	F25.1	F25.2
The number of patients with an established diagnosis	41	3	2	1	1	1

### 2.4 Non-inclusion criteria

1. Organic disease of the central nervous system;
2. Decompensation of somatic disease;
3. The period of pregnancy and lactation in women;
4. Abuse of alcohol and psychoactive substances.

The clinical and psychometric methods used in the practice of research on mental pathology were used. A single examination of patients involves:

- psychopathological and somatic examination;
- psychometric examination using standardized international scales (PANSS, FAB, NSA-4, BFCRS)

Blood sampling (8–12 ml) was carried out in vacuum tubes with heparin in a treatment room in compliance with aseptic and antiseptic rules. Transportation to the laboratory was carried out within 2 hours from the moment of collection. Blood sampling was carried out once between 8 and 9 a.m. in the clinic's treatment rooms from a cubital vein into tubes with EDTA and a gel separator, followed by centrifugation of 2000 rpm for 20 minutes. The isolated serum was stored in eppendorf type microtubes at −80, whole blood was stored at −20 until transported to the laboratory. Transportation was carried out in compliance with material safety requirements.

### 2.5 Sample preparation for MS analysis

The blood plasma in the volume of 40 μl was then brought to the final volume of 160 μl by adding the solution 15 mM MOPS (4-morpholinepropanesulfonic acid sodium salt), рН 7.4.

The dry residue was restored in 500 μl of 0.1% deoxycholic acid sodium salt, 6% acetonitrile, 75 mM triethylammonium bicarbonate, рН 8.5. The protein solution was heated up at 90 °C for 10 minutes at intensive shaking (1100 rpm). After equilibration to ambient temperature, 3 mM ТСЕР (Tris (2-carboxyethyl)phosphine) was added to the denatured protein solution to restore the sulfhydryl groups of amino-acid residues of cysteine. The reaction was incubated at 45 °C for 20 minutes. For alkylation, the denatured protein solution was added with a solution of 0.2% 4-vinylpyridine in 30% propan-2-ol up to a final concentration of 0.02% (V/V). The alkylation reaction was carried out for 30 minutes at normal temperature in the lightproof place.

Enzymatic cleavage of proteins was performed using a specific trypsin protease. The protein solution was added with modified (acetylated at primary amino groups of lysine) trypsin at enzyme-to-substrate ratio as 1:50. The reaction was incubated at 42 °C for 4 hours with intermittent mixing for 3 minutes every 15 minutes. After that, the second aliquot of trypsin was added at ration 1:100 and incubated at 37 °C continued for additional 12 hours.

Upon the time expiry the enzyme reaction was inhibited by adding the formic acid up to the final concentration of 0.5%, which also caused precipitation of insoluble deoxycholic acid. The obtained suspended solids were centrifuged at 12,000 rpm at 15 °C for 10 minutes. The supernatant (approximately 550 μl) was collected and applied to Discovery DSC solid-phase columns, which were preliminary equilibrated with the solution of 2% methanol with 0.1% formic acid. After sample application the columns were washed twice with 1 ml of 0.1% formic acid solution, and then peptides were eluted from the carrier using the solution of 70% methanol with 5% formic acid in the volume of 1 ml. The collected Eliot was dried at 30 °C for 45 minutes in a vacuum. The dry residue was restored in 40 μl of 0.5% formic acid solution and transferred into vials of deactivated glass for mass spectrometry analysis [2,3].

### 2.6 Mass spectrometry protein registration

The mass spectrometric analysis of the peptide composition of plasma samples was conducted for depleted plasma samples. HPLC-MS/MS registration of peptides was carried out using high resolution mass spectrometer Q Exactive (Thermo Scientific, USA, Catalog # IQLAAEGAAPFALGDK) by chromatographic separation using Ultimate 3000 Nano-flow HPLC system (Thermo Scientific, USA, Catalog # ULTIM3000RSLCNANO). Peptides in the volume of 5 μl were applied on enrichment column PepMap C18 for 4 minutes in the isocratic flow of the mobile phase C (2% acetonitrile, 0.08% formic acid, 0.015% trifluoroacetic acid) at a flow rate of 20 μl/min. Peptides were separated using Acclaim PepMap C18 analytical column (75 mm × 150 μm, particle size 2 μm, pore size 100 A) in the nano-flow mode in the linear gradient of the mobile phase A (0.08% formic acid, 0.015% trifluoroacetic acid) and the mobile phase B (0.08% formic acid, 0.015% trifluoroacetic acid in acetonitrile) at a flow rate of s400 nl/min at initial ratio А: В as 98:2. Separation was performed in the elution gradient from 2% to 35% of mobile phase B content for 80 minutes, followed by column washing at 90% of phase B for 10 minutes with subsequent system equilibration at initial gradient conditions for 20 minutes.

Registration of peptide signal was carried out in the dependent tandem scan mode with ionization source NSI (Thermo Scientific, USA). After rescanning of precursor ions with maximum accumulation time not more than 80 ms (or maximum accumulation value 3е6) with resolution R = 70 K in the range of 420–1250 *m*/*z*, 20 sequential tandem scans were made with maximum accumulation time not more than 120 ms (or maximum accumulation value 1е5) with resolution R = 17.5 K a with fixed minimum range value (from 220 *m*/*z*) and varying maximum range value depending on the resolved charge state. Ions with charge state z = 2+ … 5+ were selected for tandem scanning using the dynamic exclusion for the duration of one half-width of the chromatographic peak. Isolation of precursor ions was performed with the width of w = ±1 Th within the range from 9 to 17 s from the peak apex for the tandem scanning. Fragmentation was performed in the high-energy activation mode (HCD – Higher-energy collisional dissociation) with rating 27% (per weight of 400 *m*/*z* and charge z = 2+) and variation per each scanning within ±15%. HPLC-MS/MS spectra in RAW format were processed in Mass Hunter version В 2.0 [2,3].
